# Sustainable Development of Industry–Environmental System Based on Resilience Perspective

**DOI:** 10.3390/ijerph17020645

**Published:** 2020-01-19

**Authors:** Xue Wan, Xiaoning Yang, Quaner Wen, Jun Gang, Lu Gan

**Affiliations:** 1 College of Architecture and Urban-Rural Planning, Sichuan Agricultural University, Dujiangyan 611830, China; maggie_sicau_1224@163.com (X.W.); yangxiaoning73@163.com (X.Y.); wenquaner9@163.com (Q.W.); 2 Ministry of Science and Technology, Sichuan Institute of Building Research, Chengdu 610081, China; gangjun@aliyun.com

**Keywords:** sustainable development, resilience, catastrophe theory, industry–environmental system, adaptive cycle model

## Abstract

The contradiction between industrial development and ecological environment pressure has been becoming progressively severe. Under this circumstance, more attention has been paid to the balance between industrial economic development and environmental deterioration and resource consumption. Thus, this study takes the development of industry and ecological environment change as an interactive system consideration, and comprehensively evaluates the changes of the industrial–environment system on resilience perspective with innovation. Accordingly, this paper establishes a comprehensive evaluation model. The Environmental Performance Index (EPI) and Industrial Structure Entropy (ISE) were applied to analyze the current environment pressure and industrial conditions. Then, the catastrophe theory was used to evaluate the reasonably established index system for the impact of various factors in the industrial–environment system on the resilience change. Next, the adaptive cycle model was used to analyze the evaluation results and reveals the dynamic change law of the system in the resilience range. Finally, Chengdu was selected as the research area to verify the validity of the whole study. It was found that the resilient change process of Chengdu industry–environmental system accord with the four-stage theory of adaptive cycle model. The resilient level of the city was also improved during the cycle. The result of the study can be useful to future plans and decisions. What is more, understanding the characteristics of each stage will be helpful to determine the reasonable implementation time of each key factor and improve its feedback ability.

## 1. Introduction

The indispensable industrial activities in the process of economic development are directly reshaping the Earth’s environmental system [1,2]. Unhealthy industrial development modes will cause serious damage to the ecological environment, such as climate, soil, and water quality. In the past few decades, the trade-off between industrial development and environmental protection has attracted much more attention than before. The research of sustainable development management has also increased [3,4]. Reckless resource development and pollution discharge behavior has already sounded the alarm for the safety of environmental systems for human survival [5,6]. However, in order to achieve stable and rapid economic development, this unreasonable, fast-growing industrial economy pattern still exists in China. Since the reform and opening-up policy in the late 1970s, China’s rapidly-developing industries are over-reliant on energy and resource inputs and production capacity expansion [7]. Thus, China’s industry is developing into a huge source of pollutant emissions [8], which is a contradiction that urgently needs to be solved in development.

Till now, there have been many cross-over studies on industrial development and ecological environment protection. Scholars have also paid more attention to the sustainable development of the industry and environment systems from pilot studies of the environmental impact in the process of resource development. In the process of studying the relationship between industry and environment, many theories have been introduced, including resource curse theory [9], inverted U-shaped environmental Kuznets curve [10], and resilience theory [11].

The concept of resilience has been increasingly introduced into current industry–environmental system research, and has been a hot topic in many studies. For instance, Industrial Symbiosis (IS) [12,13,14], Eco-Industrial Park (EIP) [15,16], Social–Ecological System [17,18,19], and resource consumption [19,20]. These studies have gradually referred to the application of resilience trajectories to explore the interaction between regional industries and ecosystems. Resilience theory can provide a more efficient way to assess the dynamic change process with system balance and adaptability [20]. It can be used to solve the coordinated development problem between industry and environment through studying the resilient change mode of industry–environmental system under continuous mutual interference. But it has not yet received enough attention to consider the resilient relationship between industry development and environmental protection as an interactive whole system at present. Therefore, under these new challenges, this study focuses on exploring the dynamic resilience changes of the industry–environmental coupling system, and contributing to policy changes, industrial adjustment, and strategy formulation.

To analyze the dynamic resilient changes of the coupling systems under a series of external disturbances, such as environmental resource consumption, policy changes, and industrial structure adjustment, a new comprehensive evaluation method is proposed. Specifically, this paper established an evaluation index system of the industrial activities and ecological environment. Then, Environmental Performance Index (EPI) and Industrial Structure Entropy (ISE) were applied to analyze the current environment pressure and industrial conditions. The data obtained by the two calculation methods helped to initially analyze the changing trend of industry and environment, and will also serve as the data source for further operation of the evaluation model to ensure the accuracy of the evaluation results. Next, the catastrophe theory was used to evaluate the reasonably established index system of the impact of various factors in the industrial–environment system on the resilience change. The Catastrophe Progression method controlled the subjectivity of the evaluation by uncertain weight, making the evaluation results more scientific and objective. Finally, the adaptive cycle model was used to analyze the evaluation results. The characteristics of resilience dynamics conveyed by the four-phase adaptive cycle, namely exploitation, conservation, release, and reorganization [21,22], reveal the dynamic change law of the system in the resilience range. The research goals were as below:

(1) Evaluate environmental pollution, resource consuming, and industrial adjustment in a quantitative way, and give the pressure levels of different indexes in industrial–environment system. Identify resilience changing curve of industrial activities sub-system and environment subsystem by using resilience quantitative and analyze the coupled system adaptive ability.
(2) The validity of the evaluation model is verified by an empirical case. The results of the study provide guidance for the sustainable development of Chengdu and provide decision-making basis for promoting industrial development and maintaining economic and ecological balance.
(3) Master the resilient change law of the industry–environmental system, discuss and understand the characteristics of different stages, and explore the appropriate implementation cycle and feedback effects of various key indicators. In the period of industrial transformation and development of various cities, it contributes to policy changes, industrial adjustment, and strategy formulation.

The novelty of this study is outlined as follows: (1) Innovatively integrate the two seemingly independent development systems of industrial activities and environmental changes as a coupled system, and integrate the idea of resilience to research the resilient change process of the coupling system under the interaction; (2) a comprehensive evaluation model was established, which combined a variety of methods to reveal the dynamic change law of ecological environment caused by continuous industrial activities disturbances.

In general, the purpose of this study is to seek a new way for the sustainable development of the industry–environmental system, which can play an essential role in ensuring coordinated development between industrial economic development and ecological environmental protection.

The remainder of this paper is as follows: Section 2 describes the main problems in industry–environmental system. Section 3 describes approaches of the comprehensive evaluation framework, including the comprehensive evaluation indicators, catastrophe theory, and the adaptive cycle model. Following that, Section 4 provides a case study to validate the availability and applicability of the methods. The final Section 5 gives the conclusions and future research directions.

## 2. Background

### 2.1. Literature Review

As one of the important means to promote the rapid development of national economy, industry is often the first choice. Therefore, resource depletion and potential environmental damage have become inevitable. Because of this, many scholars began to pay attention to the study of industry pollution. Terao put forward the concept of “development and environment” and discussed the relationship between industrial policy and industrial pollution control in order to reduce the harm of heavy industrial pollution [23]. In China, the study of carbon emissions from pollution industries has sprung up like bamboo shoots [24]. With the acceleration of industrial development in major countries, the concepts of “green industry” [25] and “ecological industry” [26] emerge as the eras require in order to alleviate the severe situation of dual superposition of world economic crisis and environmental crisis. Due to various uncertain factors and dynamic changes in industrial activities, the environmental problems caused by them have not been completely solved, so there are more and more cross-studies on industrial activities and environmental protection. Recently, some scholars have tried to embed industrial activities in and confined by the environmental system and the resources it provides in order to analyze the interaction between them [27]. Thus, how to combine the dynamic system of industrial activities with ecological environment change has become the focus of current research.

In order to reduce the limitation of resources and environment, many explorations have been made on the sustainable development path of industrial activities and ecological environment. These extensive research topics cover low-carbon technology and circular economy [28,29], green utilization of natural resources [30], environmental health appeals [31], identification of major pollution sources in industrial development [32], sustainable resource transformation [33,34], industry–environmental policies identification [35], resilience transitions [10,36], and so on. The methods applied in these research topics involved index evaluation, economic network analysis, life cycle analysis, and model simulation. Among these studies, the concept of resilience evaluation was gradually borrowed to account for the adaptive capacity of the coupled systems. Initially, scholars tried to use the concept of resilience to evaluate urban ability to resist disasters, and gradually started to use resilience theory to study the adaptive capacity of coupled systems [37], such as human–environmental systems [38] and social–environmental systems [39]. Unfortunately, contemporary studies have not devoted sufficient attention to the resilience evolution of the industry–environmental system.

The industry–environmental system is an integrated system to recognize industrial development and environmental protection. Nevertheless, much research is not comprehensive: Some researches preferred to focus on only one or two aspects of the problems. For example, research on industrial wastewater pollution evaluation [40], efficiency evaluation of industrial system cleaner production and waste disposal [41], and industrial eco-efficiency indicators have been established [42,43]. Meanwhile, from the perspective of environmental protection, scholars studied ecological environment evaluation on all kinds of perspective [44], and analysis of the interactions among China’s economic growth and its energy consumption, air emissions, and air environmental protection investment [45]. However, industrial activities are dynamically changed, and environmental changes are an effective response to industrial adjustment. It is even more necessary to study the changes of industrial activities and the ecological environment as a coupling system. Only clarifying environmental system change laws under the continuous interference of industrial activities and the restrictive effect of environment on industrial development, it can provide a scientific basis for the sustainable development of the industry–environmental system. Therefore, this article attempts to study the interaction law between industrial and environmental systems from the perspective of resilience.

### 2.2. Research Framework

**Figure 1:**
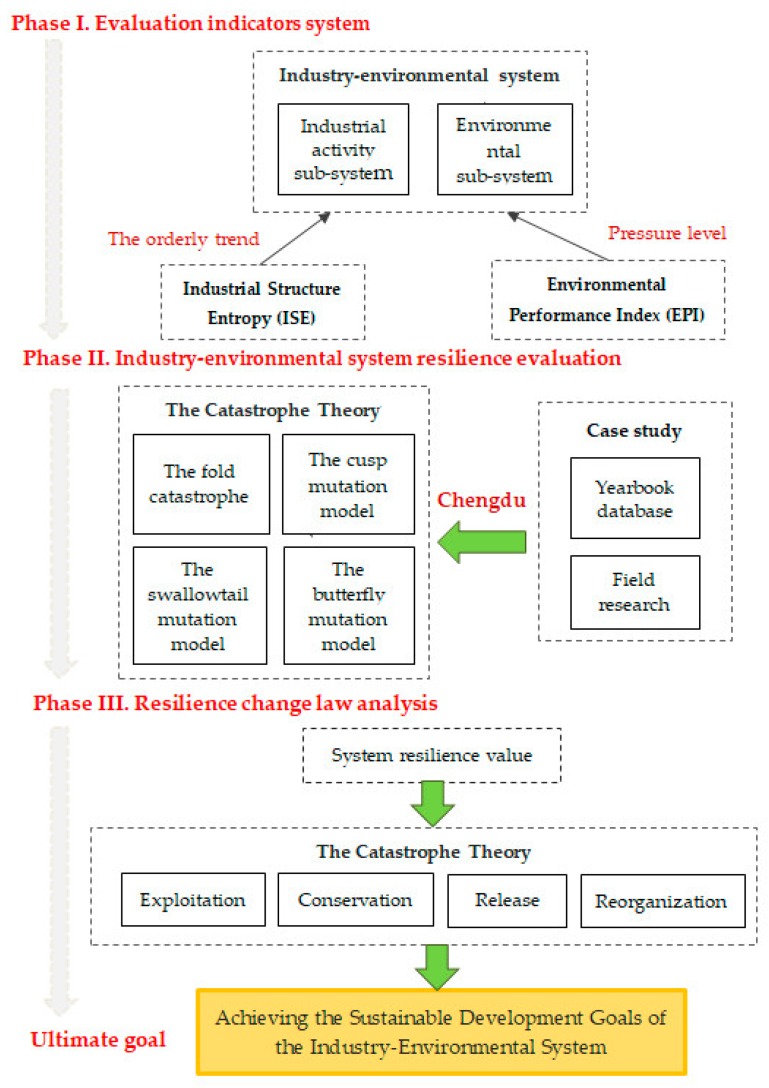
The flow chart of the research. Phase I: Establish a resilient evaluation index system, and use the Environmental Performance Index (EPI) and Industrial Structure Entropy (ISE) to show the pressure level of the environment system and the orderly trend of industrial activities in the indicator. Phase II: Calculate the resilience value of each index by using the catastrophe theory, and provides a basis for subsequent analysis. Phase III: Analyze the law of resilient change of the system through the adaptive cycle model, and apply it to the choice of future development.

This paper mainly focuses on the coupling law of industry–environmental system and look forward to finding a new way for the sustainable development of the industrial economy. Since there are respective interests of the two major themes of industrial development and environmental protection, this paper chooses to conduct a resilient evaluation of the entire industry–environmental system from the resilient perspective. The catastrophe theory and the adaptive cycle model were used to analyze how to find a balance between the industry and the environment. Therefore, establishment of comprehensive evaluation model considers the contribution of various factors in the industrial activity sub-system and the environmental sub-system to the resilience. The framework of the comprehensive evaluation model is summarized in Figure 1 with the following three research phases.

## 3. Materials and Methods

### 3.1. Evaluation Index System

This section describes the integrated resilience evaluation for industry–environmental system. Combined with the research of this paper, indicators selected from two aspects of environmental response and industrial activities in some related literatures. Table 1 briefly reviews the literature and selects the most common influencing factors for resilience evaluation of industry–environmental system.

Industry–environment integration is a highly complex system that involves the impact of industrial activities on the environment and the restrictions of environment and resources on industrial development. Based on previous studies, selecting appropriate indicators should meet the following criteria: (1) The selected indicators can be widely used in this situation; (2) the selection process should combine regional yearbooks with field research. As shown in Table 1, the most common cited indicators are economy, pollution, land use, and resource consumption. However, some indicators are often ignored, such as production and treatment. These factors still play a significant role in industry–environmental system resilience evaluation. Based on the brief literature review in Table 1 and indicators selection criteria, six main secondary indicators are selected from the industrial activities and environmental sub-systems, which are derived from seventeen primary indicators. These indicators reflect the overall situation as comprehensively as possible. At the same time, this paper will use the EPI to analyze environmental press and the ISE to analyze the orderliness of industrial structure. Therefore, the selection of indicators also considers the applicability of two index calculations. Accordingly, a comprehensive evaluation index framework is established to determine the resilient level of system with the semi-quantitative and semi-deterministic analysis. They represent the interaction between the disturbance of industrial activities and the environmental systems, including economic development, industrial structure, resource consumption, environmental pollution, and waste discharge. The detailed index system is shown in Table 2.

#### Data Standardization

Since indicators have different units, they need to be standardized to eliminate the impact of different dimensions. This also meets the requirements of the catastrophe modeling approach [38,39]. All values are normalized to values between 0 and 1 by Equations (1) and (2). The choice of equation needs to consider the positive and negative effects of the indicator. The benefit index, that is, the larger the value, the better the positive index formula is used, (Formula (1)); otherwise, the cost index uses the negative exponential formula (Equation (2)).

(1) $$\mathrm{Positive}\;\mathrm{indicator}:x_{ij}^{\prime }\;=(x_{ij}-min\{x_{j}\})/(max\{x_{j}\}-min\{x_{j}\})$$

(2) $$\mathrm{Negative}\;\mathrm{indicator}:x_{ij}^{\prime }=(max\{x_{j}\}-x_{ij})/(max\{x_{j}\}-\{x_{j}\})$$

$x_{ij}$ is the value of indicator *j* in year *i*, *max*{$x_{j}$} is maximum value of indicator *j*, *min*{$x_{j}$} is the minimum value.

### 3.2. Environmental Performance Index

The Composite Index of Environmental Performance (CIEP) was developed by the World Health Organization (WHO), based on the driving–force–pressure–state–exposure–effect–action (DPSEEA) methodology proposed [51]. The EPI is jointly developed by the Yale Center for Environmental Law & Policy (YCELP) and the Center for International Earth Science Information Network (CIESIN) at Columbia University, in collaboration with the Samuel Family Foundation and the World Economic Forum [52]. The CIEP and the EPI are two composite indexes commonly that are used to quantitatively measure ecological quality or impact and used in many studies that often accept innovation and improvement [53]. Those indicators can be used to guide the operation of all subsequent steps. This paper needs to select the most suitable one to represent the pressure level of environmental indicators and reflect the resilience level of the environmental system. According to Neves Almeida’s [54] analysis of the possible differences between the CIEP and EPI indices, the model compares the pros and cons of the two. Using the two EPI objectives of environmental health and ecosystem vitality, CIEP is explained by two models and ten policy categories of EPI are adopted. The third model uses EPI as the dependent variable and the independent variables are the following five CIEP dimensions: driving force, pressure, state, effects, and actions.

*CIEP _it_* = *α* + *β*_1_*eh_it_* + *β_2_ev_it_* + *φ_i_* + *φ_t_* + *ε_it_* (Model 1)

*CIEP _it_* = *α* + *β*_1_*eheh_it_* + *β*_2_*ehair_it_* + *β*_3_*ehwater_it_* + *β*_4_*evair_it_* + *β*_5_*evwater_it_* + *β*_6_*evbh_it_* + *β*_7_*evag_it_* + *β*_8_*evforest_it_* + *β*_9_*evfish_it_* + *β*_10_*evclimate_it_* + *φ_i_* + *φ_t_* + *ε_it_* (Model 2)

where, *α* is the constant; *i* is the individuals (countries); *t* in the time (year); *eh* is environmental health and *ev* is ecosystem vitality; *φ_i_* and *φ_t_*, are the dummies to measure the individuals and time effects, respectively. The *ε_it_* is the random effect.

*EPI _it_* = *α* + *β*_1_*df_it_* + *β*_2_*p_it_* + *β*_2_*s_it_* + *β*_2_*ef_it_* + *β*_2_*a_it_* + *φ_i_* + *φ_t_* + *ε_it_* (Model 3)

where, *α* is the constant; *i* is the individual countries; *t* in the time (year); *df* is the driving force, *p* is the pressure, *s* is the state, *ef* is the effects and *α* is the actions, *φ_i_* and *φ_t_* are the dummies to measure the individual countries and time effects, respectively. The *ε_it_* is the random effect.

According to the difference in comparison results, the CIEP model uses multiple imputations to complete the missing values, but the EPI does not use any imputation methods. The EPI also includes environmental policy factors. Moreover, the EPI can be calculated only with ecological index data and GDP value, and conforms to the rules of ecological tools used by the decision-maker to formulate environmental policies [54]. Therefore, combined with the effectiveness and practical convenience of this study, EPI is more suitable to reflect the pressure level of resource consumption and pollutant emission in industrial activities [33]. It is easier for policy makers to refer to environmental policies. Therefore, this paper chooses EPI to reflect environmental pressure. The calculation process can be expressed as the following formula:

(3) $$EPI=(x_{i}/g)(X_{i}/G)$$

where *x_i_* is the total consumption of environmental factor *i* in the study area; *X_i_* is the total consumption of environmental factors *i* in the country; *g* is the GDP of the study area; *G* is the GDP of the entire country. The smaller the EPI, the higher the utilization level of environmental factors or the lower the environmental pressure of the study area [33].

### 3.3. Industrial Structure Entropy

Frequent economic activities have brought increasing pressure on the ecological environment. The rationality of industrial structure has important impact on the ecological environment [46]. Therefore, it is necessary to consider the influence of industrial structure in the industry–environmental system. Industrial Structure Entropy (ISE) is selected to describe the state and degree of industrial structure system evolution [55].

Entropy is a physical quantity that indicates the degree of disorder of molecular states. It was later that Shannon firstly introduced the definition of information entropy in the information theory, which can be used to demonstrate the probability and statistics methods to prove the chaotic state of any system [56]. Thus, the industrial structure entropy is used in the industrial structure to describe the state of industrial structure system evolution. The industrial structure is stable, the uncertainty is small, and the entropy is small. Conversely, if the industrial structure is chaotic, the uncertainty is large, and the entropy is large [57]. Therefore, by using the Shannon–Weaver formula [58], it is expressed as:

(4) $$H=-\sum_{i\;=\;1}^{n}P_{i}\;\times \;lnP_{i}$$

where: *H* represents the ISE; *n* represents the number of industry; *P_i_* represents the proportion of the output value of *i* industry in the output value of all *n* industries in the region. A larger *H* indicates that the industrial structure is more disorderly, discrete, and diverse [33].

### 3.4. Catastrophe Theory

Any movement in nature or human society has always been in a stable and unstable state. Under the influence of tiny accidental interference factors, the steady state can still recover. But when the system is too strong to change, absorb, and adapt, the system will enter another new stable state or another state. The range of effective adjustment is the purpose of elastic evaluation. A range of approaches have been applied to resilience assessment such as quantification based on indicators (quantitative) [59] and framework (qualitative) [60]. Theoretical resilience frameworks are built on collections of concepts or ideas. The framework-based approach mainly guides people to think about how to quantify resilience [61]. In recent years, more and more scholars have made achievements in the quantitative research of resilient change. The following Table 3 has some typical quantitative models in related fields.

Catastrophe theory can directly deal with discontinuity without linking any specific internal mechanisms; therefore, it is particularly applicable for the study of systems with unknown internal functions. The study of industry and environment as a whole system is a new exploration, so the resilience change of industry–environment system is unknown. Based on the above resilient quantification methods, it is the most suitable theory to analyze.

The catastrophe theory studies the phenomena and laws from a stable configuration to another stable configuration. It describes the evolution of form, especially for the analysis of sudden dynamic changes caused by gradual changes in force [65,66]. The solution process combines the fuzzy theory to generate the mutated fuzzy membership function by evaluating the multi-level contradiction decomposition of the target. It then normalizes the quantitative operation through the normalization formula. Finally, the normalization function is a parameter that obtains the total membership function and analyzes the evaluation objectives.

The method is characterized by not weighting the indicators, but taking into account the relative importance of each indicator. Thereby, it can reduce subjectivity without losing scientific and rationality through simple and accurate calculation in a wide application range. At the same time, the theory can detect changes in the resilient variation between industrial and environmental randomness with different equilibriums, and can solves the adaptability of the resilient system. Furthermore, the results of catastrophe theory represent the time-dependent transformation of the coupled systems rather than simply increasing values of all indicators [20]. Considering these two key features of catastrophe theory, this study chose it as a method of industry–environmental system resilient calculation.

There are seven basic mutation models, namely, folding mutation, cusp mutation model, swallow tail mutation model, butterfly mutation model, hyperbolic umbilical cord, elliptical umbilical cord, and parabolic umbilical cord. However, the commonly-used mutation progression methods are fold catastrophe, cusp mutation model, swallowtail mutation model, and butterfly mutation model.

The fold catastrophe is:

*f(x)* = *x*^3^ + *ax* (5)

The cusp mutation model is:

*f(x)* = *x*^4^ + *ax*^2^ + *bx* (6)

The swallowtail mutation model is:

(7) $$fx=\frac{1}{5}x^{5}+\frac{1}{3}ax^{3}+\frac{1}{2}bx^{2}+cx$$

The butterfly mutation model is:

(8) $$fx=\frac{1}{6}x^{6}+\frac{1}{4}{ax}^{4}+\frac{1}{3}{bx}^{3}+\frac{1}{2}{cx}^{2}+dx$$

where *f(x)* represents the potential function of the state variable *x* of a model, and the coefficients *a*, *b*, *d*, *c* of the state variable *x* represent the control variables of the state variable. If an indicator can only be decomposed into two sub-indicators, it is a cusp catastrophe model; if there are three sub-indicators, it is a swallowtail mutation model; if there are four sub-indicators, a butterfly mutation model.

The normalization formula is solved by the potential function *f(x) = x*^4^ + *ax*^2^ + *bx* of the cusp mutation. Let *∂f(x)/∂x = 0*, the equation of the critical point of the *f(x)* can be got:

*f′(x)* = *4x*^3^ + *2ax* + *b* = 0 (9)

Let *∂f′(x)/∂x = 0*, the equations of all singular point sets the *f(x)* can be got:

*f″(x)* = *12x*^2^ + *2ax* = 0 (10)

Combine (9) and (10), eliminate *x*, and get a divergence equation: *a = −6x*^2^, *b = 8x*^3^, and then get:

$$x_{a}=\sqrt{-\frac{a}{b\;}},x_{b}=\sqrt[{3\;}]{\;\frac{b}{8}\;}$$

Using this as a normalization formula will result in inconsistent values of *x*, *a*, and *b*, and will also make the value range of (0,1) of the utility function and fuzzy membership function inconsistent, so control the value of the variable *x* and the variables *a, b* in the interval [0,1]:

Since *x_a_* = $\sqrt{-\frac{a}{6}}$, when *x_a_* is in the interval [0,1], the corresponding value range of *a* is also in the interval [0,1]. Then the value of *a* needs to be 6 times smaller, therefore *x_a_* = $\sqrt{a}$. The same reason, let *b* also shrink 8 times, then *b* will also be in the interval [0,1], *x_b_* = $\sqrt[{3}]{b}$.

So, the four catastrophe model normalization formulas are is derived by the bifurcation equation:

The fold catastrophe is:

(11) $$\;X_{a}\;=\;\sqrt{a}$$

The cusp mutation model is:

(12) $$X_{a}\;=\;\sqrt{a},\;X_{b}\;=\;\sqrt[{3}]{b}$$

The swallowtail mutation model is:

(13) $$X_{a}\;=\;\sqrt{a},\;X_{b}\;=\;\sqrt[{3}]{b},\;X_{c}\;=\;\sqrt[{4}]{c}$$

The butterfly mutation model is:

(14) $$X_{a}\;=\;\sqrt{a},\;X_{b}\;=\;\sqrt[{3}]{b},\;X_{c}\;=\;\sqrt[{4}]{c},\;X_{d}\;=\;\sqrt[{5}]{d}$$

Two important principles must be considered in the fuzzy comprehensive evaluation, when using the catastrophe progression method. If there is no obvious correlation between the control variables of the same project variables (such as *u*, *v*, and *w*), then the control variables are called non-complementary variables (such as *u*, *v*, and *w*), otherwise the control variables are called complementary variables (such as *u*, *v*, and *w*). When the catastrophe membership function value is calculated, as for non-complementary variables, the *x* is the minimum value of the corresponding control variable. As for complementary variables, the *x* is the average value of the corresponding control variable, such as *x* = (*x_u_ + x_v_ + x_w_*)/3.

According to the calculation process of the catastrophe progression method, a normalized formula is adopted to calculate the catastrophe membership function value gradually until the comprehensive catastrophe membership function value is obtained.

### 3.5. Adaptive Cycle Model

**Figure 2:**
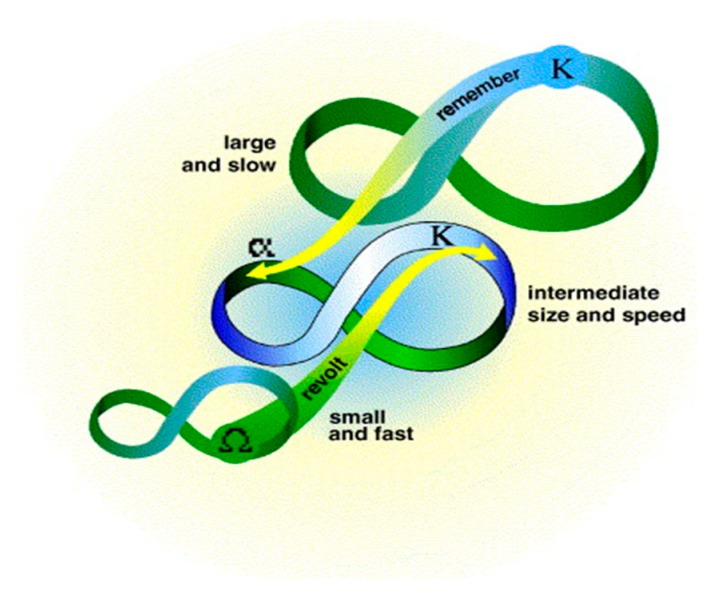
Adaptive cycle in resilience interpretation.

The final step is to instantiate the resilience values that calculated by using the catastrophe progression method. Adaptive cycle model is an important model, and has a wide range of applications in resilient analysis [33,38,67]. Adaptive loop provides a systematic approach that uses four phases to illustrate the resilience changes at different stages and reveals the adaptive ability of adaptive loops [21]. They include “exploitation (*r*)” (with a significant increase of resilience), “conservation (*K*)” (accumulation, monopolization, conservation of structure and resilience tends to decline), “release (*Ω*)” (rapid collapse or release phase of creative destruction) and “reorganization (*α*)” (renewal and reconstruction phase during the resilience tends to increase), which is shown in Figure 2. It must be emphasized that the adaptive cycle is neither constant nor absolute, but the most diverse. Understanding the phases of these cycles, their temporal and spatial extent and succession, it is important to identify the points at which the system can accept positive changes and the points at which the system is susceptible or likely to be affected by regime changes [68]. These four phases can be divided into two cycles: The “fore-cycle” (from the exploitation phase to the conservation phase) and the “back-loop” (from the release phase to the reorganization phase) [49]. The conversion of adaptive loops in two cycles can be used for the adaptation and conversion capabilities of the surveillance and navigation systems. When the industry–environmental system fails to resolve the “back-loop” phase, the system’s adaptability will be lost, which may lead to the system’s resilience collapse. The “fore-cycle” phase is the time to implement the new policy, which represents the state of the industry–environmental development required [22,69].

## 4. Case Study

In order to verify the effectiveness of the proposed evaluation model, this paper used the data of Chengdu to calculation, the city located in central Sichuan Province. The test aimed to illustrate how the proposed comprehensive evaluation model can be applied to the analysis of industry–environmental system resilience and future plan.

### 4.1. Research Case Area

**Figure 3:**
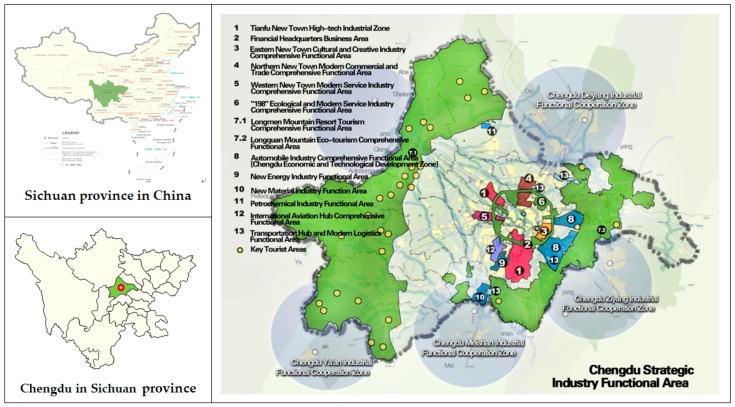
Location of study area.

As a national regional central city, Chengdu is the center of the economy, technological innovation and financial trade in Southwest China. At the same time, in recent years, Chengdu put forward a development goal: To build inland open economic highlands and a Park City (PC) with ecological value. PC is the sublimation of the traditional urban planning concept under the new era, and it is a new mode of sustainable development of urban construction. At this point, Chengdu has become the first city to mention the concept of PC. The location of the study area is shown in Figure 3.

For a long time, the industrial economy has been the main source of economic growth in Chengdu. However, due to the special watershed environment, Chengdu has always faced tremendous ecological pressure. Therefore, coordinating industrial development and environment issue is an inevitable problem for the future development of Chengdu. In order to promote the construction of the “Made in China 2025” pilot demonstration city, Chengdu has proposed to focus on the development of five pillar industries about electronic information, automobile manufacturing, food and beverage, equipment manufacturing, and biomedicine. Meanwhile, it also forms an industrial functional cooperation zone with neighboring cities in the economic zone, and neighboring cities such as Ya’an promote the integration of industry and environment in the form of vigorously developing tourism. While developing emerging industries, the impact of the rapid development of traditional industries on ecosystems cannot be ignored. The problem of industrial pollution problems and the effective utilization of resources are still urgent problems to be solved. Therefore, it is an inevitable choice to choose Chengdu as the research area to analyze the elastic relationship between Chengdu’s industry and environmental variables. The results of Chengdu case study will contribute to the sustainable development of other ecological cities in China.

### 4.2. Data Acquisition and Processing

The data of industrial activities, environmental pollution, and resource consumption used in this paper are from 2000–2018 Chengdu Statistical Yearbook, Sichuan Statistical Yearbook, China Statistical Yearbook, China Energy Statistical Yearbook, and Statistical Communique of Chengdu’s National Economic and Social Development. The raw data for these indicators are all from the annual statistical yearbooks. In the evaluation application process, the first-hand data obtained from the statistical yearbook is usually directly used for analysis, but many data are not blindly pursued the maximum or minimum value is the optimal value. In a complex system, the choice of the optimal value needs to weigh the multi-interest and take the optimal value under comparison. At the same time, some indicators are not easy to quantify directly, so the data of some indicators are processed with EPI and ISE in this study. The final data after calculation and analysis are selected as the data of resilient assessment analysis.

#### 4.2.1. Data Processing Results Analysis of EPI

Four resource consumption indicators and four pollution discharges indicators from 2000 to 2017 are calculated by EPI index based on the Equation (3). These indicators are selected to reveal the environmental pressure since 2000. Because of the “treatment” indicators are mainly used to reflect the improvement of technical level and environmental awareness, they are abandoned here. The calculation results roughly reflect the changes in the environmental system of Chengdu since 2000. The pressure on most environmental factors is decreasing. The following Table 4 is the calculation result for the eight indicators represented by EPI in the environment sub-system.

**Figure 4:**
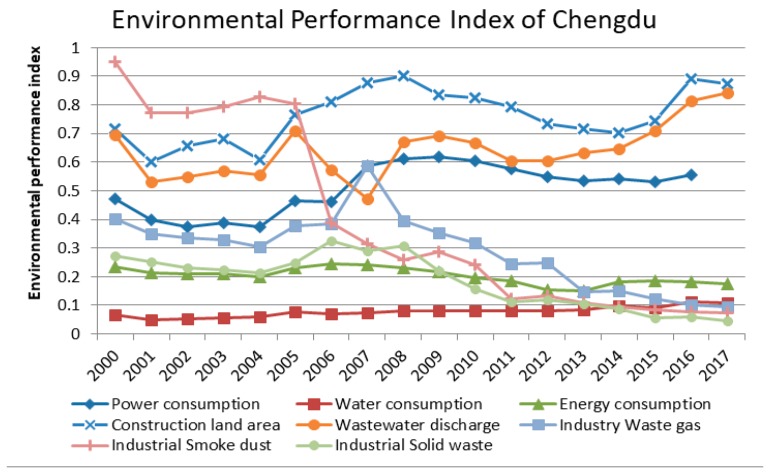
EPI of eight environmental factors in Chengdu from 2000 to 2017.

The performance indicators of eight environmental factors of Chengdu from 2000 to 2017 are based on the calculation results of Equation (3) as shown in Figure 4.

Since 2000, the environmental pressure of the three indicators of industrial pollution emissions has decreased significantly, indicating that environmental protection policies and industrial transformation are gradually releasing the pressure of environmental pollution. The performance of industrial smoke dust and industrial waste gas is the most obvious. However, the EPI of the three environmental factors, that is, electricity consumption, sewage discharge, and construction land fluctuated greatly. In the meantime, there was a slight upward trend, which indicates it is necessary to improve the economic utilization of electricity and land resources, and strengthen the governance of wastewater discharge problems. This situation may be the result in rapid urban expansion and the vigorous development of high-tech industries. Simultaneously, during the research period, the EPI of energy consumption was relatively stable and slightly decreased, indicating that the adjustment of industrial structure is gradually reducing the dependence of economic growth on resource-dependent industrial development, such as heavy industry. What is more, it also reflects the higher level of energy resource utilization. Due to the superior environmental location of the Chengdu Plain, water resources have remained stable and low for a long term. Also, abundant water resources greatly promoted Chengdu’s industrial development.

#### 4.2.2. Data Processing Results Analysis of ISE

**Figure 5:**
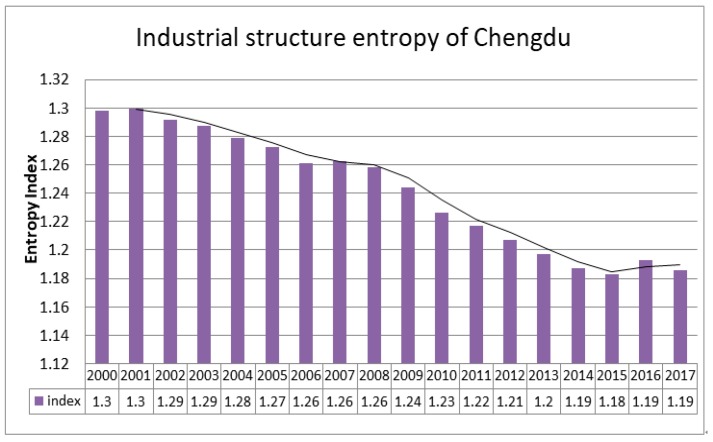
ISE in Chengdu from 2000 to 2017 and the two-period moving average curve reflects the trend of ISE.

The ISE is calculated by the four types of indicators, that is Chengdu’s primary industry output value, secondary industry output value, tertiary industry output value, and industrial gross output value from 2000 to 2017. The calculation results reflect the changes in the industrial structure adjustment of Chengdu. In the past 20 years, the industrial structure of Chengdu has become more and more stable. According to the calculation results of ISE based on the Equation (4), the change index of industrial structure from 2000 to 2017 is shown in Figure 5.

The ISE of Chengdu in the past 20 years is generally concentrated between 1.18 and 1.3 showing a certain stage characteristics. The ISE value fluctuated and declined in the period from 2000–2007. It continued to decline rapidly from 2008 to 2015 and has rebounded slightly since 2015. Overall, the ISE value has decreased significantly, and the industrial structure has been continuously improved. In recent years, after the adjustment of Chengdu’s industrial structure, it has changed the situation of the industrial upgrading lagging behind economic development. The industrial structure has undergone major changes, which have gone from “two, three, one” to “three, two, one”. The industrial structure of Chengdu has been transformed into a new industrial structure dominated by industry and modern service industries. It is further alleviating the pressure on the environmental system and providing a breathing space for the rebound of the industry–environmental system.

### 4.3. Analytic Results

According to the index system established in the previous section, the resilience value of the factors represented by each index in the layer, and the comprehensive resilience value of the environmental and industrial sub-systems are all calculated by the catastrophe progression method. The indicator data used in the evaluation are derived from statistical yearbooks and data calculated by EPI and ISE. According to the number of control variables, each layer index is selected corresponding to the appropriate catastrophe model, and the formula corresponding to the model is selected in Equations (12)–(14) for calculation.

Next, the detailed calculation procedure for the catastrophe progression method is provided with some simple example using data from Chengdu.

(1) Calculating the membership degree for criteria layer (B) and use its corresponding indicators (C) as control variables, and in accordance with complementary principle.

Cusp model for B3 and B6:

$$x_{B3}\;=\;({x_{C37}}^{1/2}\;+\;{x_{C38}}^{1/3})/2\;=\;(0.787\;+\;1.00)/2\;=\;0.893\;x_{B6}\;=\;({x_{C616}}^{1/2}\;+\;{x_{C617}}^{1/3})/2\;=\;(0.571\;+\;0.00)/2\;=\;0.285$$

Swallowtail model for B1, B2 and B4:

$$x_{B1}\;=\;({x_{C11}}^{1/2}\;+\;{x_{C12}}^{1/3}\;+\;{x_{C13}}^{1/4})/3\;=\;(0.453\;+\;0.357\;+\;0.097)/3\;=\;0.302\;x_{B2}\;=\;({x_{C24}}^{1/2}\;+\;{x_{C25}}^{1/3}\;+\;{x_{C26}}^{1/4})/3\;=\;(0.468\;+\;0.822\;+\;0.00)/3\;=\;0.784\;x_{B4}\;=\;({x_{C49}}^{1/2}\;+\;{x_{C410}}^{1/3}\;+\;{x_{C411}}^{1/4})/3\;=\;(0.366\;+\;0.905\;+\;0.880)/3\;=\;0.717$$

Butterfly model for B5,

$$x_{B5}\;=\;({x_{C512}}^{1/2}\;+\;{x_{C513}}^{1/3}\;+\;{x_{C514}}^{1/4}\;+\;{x_{C515}}^{1/5})/4\;=\;(0.630\;+\;0.721\;+\;0.00\;+\;0.717)/4\;=\;0.517$$

(2) Calculating the membership degree for sub-system (A) and use its corresponding criteria layer indicators (B) as control variables. The industrial activity sub-system is calculated according to the principle of complementarity, and the environment sub-system is based on the principle of non-complementarity.

Cusp model for A1:

$$x_{A1}\;=\;({x_{B1}}^{1/2}\;+\;{x_{B2}}^{1/3})/2\;=\;(0.550\;+\;0.755)/2\;=\;0.652$$

Butterfly model for A2:

$$x_{A2}\;=\;\min\{{x_{B3}}^{1/2},\;{x_{B4}}^{1/3},\;{x_{B5}}^{1/4},\;{x_{B6}}^{1/5}\}\;=\;\min\{0.845,\;0.895,\;0.848,\;0.778\}\;=\;0.778$$

(3) Calculating the catastrophe membership degree for industrial–environment system resilience, and in accordance with non-complementary principle.

Butterfly model for A:

$$x_{A}\;=\;\min\{{x_{A1}}^{1/2},\;{x_{A2}}^{1/3}\}\;=\;\min\{0.743,\;0.920\}\;=\;0.743$$

The all calculation results are drawn into the following three line graphs.

#### 4.3.1. Environmental Sub-System

**Figure 6:**
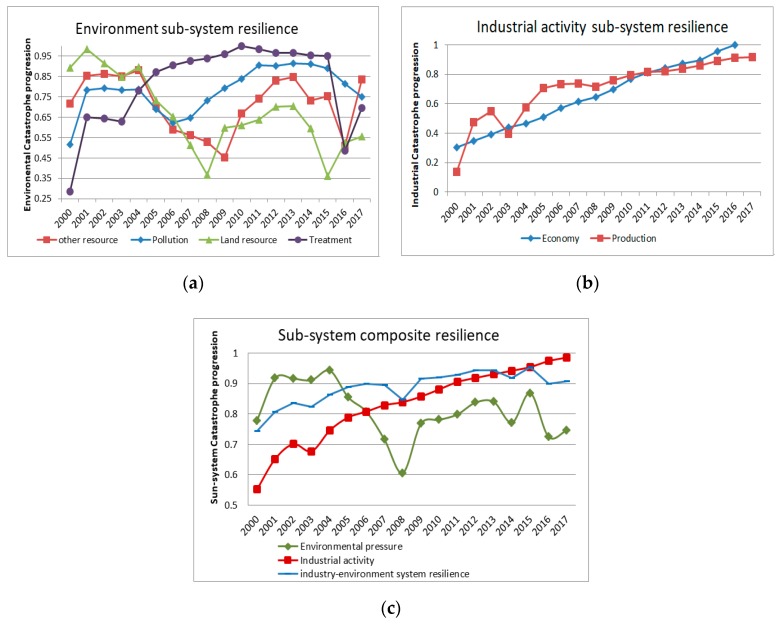
(**a**) Changes in the resilience values of the four main indicators of the environmental sub-system; (**b**) changes in the resilience values of the two main indicators of the industrial activity sub-system; and (**c**) changes in the composite resilience values of the environmental sub-system, the industrial activity sub-system and the industry–environmental system. The resilience value from 0 to 1 indicates ascending adaptive capacity.

The line chart of Figure 6a shows vividly that from 2000 to 2017, the resilience of all environmental factors experienced a typical transition period. Two distinct points of change are 2008–2009 and 2015–2016. Overall, there has been a positive change in environmental resilience resulting in a constant change in adaptive capacity. In addition to land resource, the other environmental factors are shown more resilient than the beginning of the research period. Especially in pollution and treatment, the rising trend of adaptive curve is more significant. The stronger the resilience of various factors in the environmental sub-system, the lower the resource pressure and the stronger the adaptability.

It can be seen from Figure 6a that the adaptive curves of the four indicators of the environmental sub-system have three distinct fluctuation periods. The adaptive curve of environmental resources demonstrates its vulnerability. Their release phase can be observed: The adaptation curve of land resources declined rapidly in 2004–2008 and 2013–2015, and the adaptability curves of other resources also showed in almost the same period in 2004–2009, and 2015–2016 is a clear downward trend. This is a positive environmental response to the dynamic changes of industrial activities. Chengdu began to promote the policy of Urban and Rural Industrial Development Integration in 2003, which promoted the conversion of a large amount of rural land into construction land. According to the Statistical Yearbook data, it can be found that the growth rate of Chengdu’s resource consumption level was significantly faster than the national level during the period of the adaptability curve declined. At the same time, the bottom of land resource resilience in 2008 was also due to the 2008 Wenchuan earthquake, which caused the massive destruction of urban land resources. The orderly construction after 2008 has restored land resource resilience. What is more, high-tech industries such as the IT industry have gradually become Chengdu’s iconic industries from 2009, which has also eased the dependence of Chengdu’s economic development on resource consumption. As a result, the adaptive curve shows an upward trend. The policy of Green Development and Economic Transformation in 2012 has brought a new round of resilient changes. Environmental pollution and treatment are experiencing increasing resilience. The adaptive curve of environmental treatment has emerged for the first time in 2015–2016. The downward trend of pollution pressure and governance pressure helps maintain a healthy environment which could support the industry–environmental system functional and long-lasting system development.

The comprehensive resilience of the environmental sub-system is affected by four indicators and the resilience is constantly changing. As can be seen from Figure 6c, the resilience of resources such as land, water, electricity, and energy rapidly decline in 2004–2008, and 2013–2016 has also led to the adaptability of the environmental sub-system decline. However, in general, the resilience value of the environmental sub-system has improved compared with 2000 indicating that the local industry–environmental system has recovered and improved its adaptability for resource consumption and environmental pollution. In addition, the vulnerabilities and fluctuations of observed variables should be addressed to answer the challenges of limited resources and environmental risks during industrial transformation. Therefore, it is necessary to formulate positive and effective policies to solve future industry–environmental system’s development problems in order to improve adaptive capacity.

#### 4.3.2. Industrial Activity Sub-System

The industrial activity sub-system is different from the fluctuation of the environmental sub-system. The resilience value of the industrial sub-system is shown in Figure 6b as an increasing trend. Only the indicators show that the industrial structure suddenly dropped in 2002–2003. During the period, Chengdu industrial restructure was carried out for the first time, and the major strategic decision of structural adjustment of the eastern suburb industrial zone was implemented. The trend then quickly turn a reorganization phase in which the resilience value continued to increase steadily. The resilience of the industrial activity sub-system is also affected by the industrial structure indicators, and there was a small drop between 2002 and 2003, which was also due to the changes in agricultural restructuring. The industrial structure adjustment gradually reduced the proportion of the primary industry. Among these influencing factors, the industrial structure is the key dominant factor of the industrial activity sub-system when the system is in the lowest or highest resilient state. Overall, the industrial activity sub-system has experienced a relatively stable upgrade and transformation, and the adaptability has been significantly improved.

Policy makers and environmental managers of regional industrial development are very interested in understanding the economic effects of industrial restructuring and the positive impact on the environmental system. Understanding the structure, direction and innovation of the industry and the interaction of the environment can help to develop viable new development plans to develop emerging alternative industries and minimize environmental burdens. At the same time, the exploration of other aspects of industrial sustainable development will also provide us with valuable experience [70,71].

#### 4.3.3. Resilience Change in the Industry–Environmental Systems

When catastrophe theory is applied to the quantitative calculation, the calculation equation is the normalization formula. Therefore, the calculated resilience value is usually very high, and the obtained resilience values are very close to each other [72]. The numerical value of the resilience value is not suitable as a basis for evaluating the resilience change. Thus, through the K-means clustering analysis in the SPSS software, the calculated composite resilience values of the industry–environmental system are clustered and analyzed with five different levels: Non-resilience, low-resilience, resilience, mid-resilience, and high-resilience. This makes it easier to judge the four adaptive cycles of resilience. The results of cluster analysis of SPSS are shown in Table 5 below. Table 5 shows the range of values for different resilience levels from low (level 1) to high (level 5).

**Figure 7:**
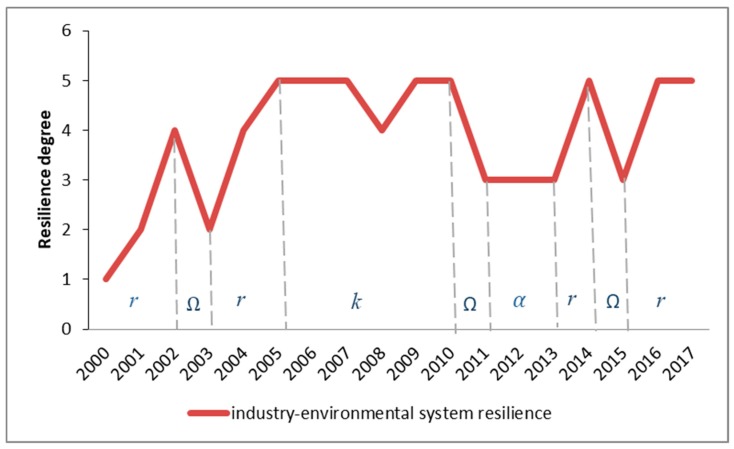
Resilience grades changes of the industry–environmental system.

According to the K-means clustering analysis, the line chart of the industry–environmental system resilience from 2000 to 2017 is drawn as Figure 7 below. The variation of the line chart uses the four stages of the adaptive cycle model to analyze the changes in resilience.

It can be seen from Figure 7 that the comprehensive resilience grades curve of the industry–environmental system presents a wave-like upgrade, which shows the process of adaptive cyclic transformation of the coupled system. The resilience grades are in the “exploitation” stage from 2000 to 2002. The resilience grades show a rapid growth and peaked in 2002. Then it enters the “release” stage, and the resilience grades dropped sharply to level 2 in 2003. After that, it entered the “exploitation” stage that resilience grades rapidly increased to level 5. The subsequent period of 2005–2010 is a long-term “conservation” stage with resilience accumulation, showing a downward trend. After the “conservation” stage, the resilience grades of the industry–environmental system go through the “release” and “reorganization” stages, complete the “back-loop” phase and the adaptability improved. The end of the “back-loop” phase also marks the beginning of a new round of policy development, and indicates the regional development goals are in line with the future development requirements of industrial activities and environmental system. Finally, the industry–environmental integration system enters the next cycle in 2013, and the resilience grades experience a sharp increase and decline again. It is currently in the “conservation” stage, and the industrial–environment system returns to the highest level of resilience.

The curve shows that since 2000, Chengdu’s development goals and direction adjustment behavior have carried out within its own flexibility, and the city resilient level is also improved in the cycle, it ensures the adaptability of the industrial environment system itself. Starting with the adaptive cycle model, the “exploitation” and “reorganization” phase is the best time point to adopt the new management strategy to enhance resilience, and the “release” stage is the most vulnerable stage. Analyze the characteristics of the four stages will help to grasp the key points of policy promotion and protection. The most recent major industrial adjustments and environmental policies announcements in Chengdu was in 2004–2010. For example, *The Guidance Catalogue of Industrial Structure Adjustment (2005)*, *the 11th five-year Plan of Chengdu Environmental Protection* in 2007, and *the 11th five-year Plan of Chengdu Industrial Economic Development* in 2008. During this period, Chengdu carried out three time industrial restructure. These all confirm the analysis of adaptive cycle model.

### 4.4. Discussion

This study modeled and described the resilience of the industry–environmental system based on catastrophe theory and adaptive cycle model. By considering industrial activities and environmental systems as a self-organizing system, this integrated approach helps to explore how the negative consequences of new initiatives in different periods can transform the system into a relatively sustainable and resilient state.

Results and analysis show that the research results are consistent with the laws of adaptive cycle model. As this study found in the above comprehensive resilience grades curve, the current overall resilience has been significantly improved. Meanwhile, the curve shows that Chengdu’s development goals and directions since 2000 have completed the “back-loop” phase, which is the guarantee of the industry–environmental system’s own adaptation and resilience. However, rapid economic development and urbanization are inevitably main sources of environmental pollution and degradation. In addition, the resilience grade of the industrial sub-system is significantly enhanced, while the resilience value of the environmental sub-system shows obvious fluctuations. It indicates that the rapid development of the economy and the continuous improvement of the industrial structure still threaten the environmental sub-system. Reasonable adjustments and updates to the development goals of the industrial–environment system are needed based on the rule of system resilience.

Adaptive cycle model believes that resilience systems must undergo cycles of exploitation, conservation, release, and reorganization. Starting from the adaptive cycle model, the exploitation phase is the best time point to adopt the new management strategy to enhance resilience. But the release phase is the most vulnerable phase. It may lead to irreversible resilience loss. Only the completion of the “back-loop” phase represents a recovery of resilience, which formed by the release and reorganization phase. Through the quantitative analysis of the catastrophe method and qualitative analysis of the adaptive cycle model, the key drivers in the resilience development of the industry–environmental system are determined. It includes industrial structure, urban development and construction, and the utilization degree of environmental resources. The study found that the industrial structure and industrial proportion of industrial activity sub-systems are the key factors for the development of industrial resilience. The control of resource consumption in environmental sub-systems is the key to future environmental governance. Environmental pollution and resource consumption caused by industrial production structure changes are the key factors for balancing the environmental resilience of coupled systems. These different dimensional changes affect the transition between different phases in the adaptive loop to varying degrees. These indicators determine the resilient state of the sub-system, the resilience trend of the industrial activity sub-system, and the environment sub-system under mutual influence. Mastering the laws of the system’s resilience cycle will really help to rationally adjust and update the sustainable development goals of the industry–environmental system. It helps to determine the reasonable implementation time of new policies and countermeasures.

The cycle model provides a reference for the next policy development, and also explains the reasonable implementation time of the relevant actions. Understand the structure, direction, and innovation of the industry and the interaction of the environment to minimize the environmental burden. The research on this system has helped to analyze the most vulnerable issues in sustainability management. Sensitive indicators have become the key control indicators for future sustainable development, and time-based strategic adjustments to ensure industry–environmental resilience.

## 5. Conclusions

In this study, EPI and ISE were used to measure the pressure level of eight environmental factors and the stability of industrial structure. Then the catastrophe theory was used to calculate the resilience value of the system and analyze the absorption and adaptability of the industry–environmental system. In addition, the adaption cycle model was used to study the resilient dynamics of the system in order to reveal the resilience change of the system. The four-stage cycle process demonstrates the process of industry–environmental system destruction and recovery, as well as the response of industrial activity sub-systems, environmental sub-systems, and integrated systems to various resilient interference factors. The comprehensive evaluation model explores the key factors, the process and the change law that affect resilient change in industry–environmental system.

According to the exploration of this paper, we can better grasp the focus of the policy adjustment and the reasonable implementation time of various measures to promote the sustainable development of the industry–environmental system. On the basis of different characteristics of four stages, the adaptive cycle model is used to discuss the suitable implementation cycle and feedback effect of each key index. In the period of industrial transformation and development in various cities, it contributes to policy adjustment, industrial adjustment and strategy formulation. Through the resilient grade change curve, we can grasp the reasonable action point of the new policy implementation and avoid the fragile period of the industry–environmental system. It provides a reference for the next policy and implementation nodes. The research of the system is helpful to understand the most vulnerable problems in sustainable management and to explore a new model for the sustainable development and construction of ecological cities such as Chengdu. The sensitive index has become the key index for the future sustainable development, and they will be the breakthrough point of the future research.

The catastrophe theory used in this paper is the most direct and convenient method to measure the resilient level in the index evaluation. Then analyze the resilient law by adaptive cycle model. The transition between stages in the adaption cycle model is a key decision point to ensure system resilience. This is a continuous but disconnected process. Further exploration of more effective tools is needed to directly assess elastic changes and obtain accurate data for each key point threshold. Exploring the laws of resilient change is only the first step need to be taken in the research of the resilience of the industry–environmental system. Further research on system threshold determination and variable range can also help to confirm the effectiveness of policy implementation and extract optimal solution.

## Figures and Tables

**Table 1:** Impact factors of industry–environmental system evaluation.

Authors	Environment Sub-System						
	Economy	Production	Industrial Structure	Land Use	Pollution	Treatment	Resource Consumption
Yang et al. [33]	√		√	√			√
Li et al. [38]	√	√	√	√	√	√	
Chen and Zhao [46]	√	√	√		√	√	
Zhou et al. [47]	√			√	√		√
Xiong et al. [48]	√				√		√
Wang et al. [49]	√		√		√	√	√
Huang et al. [42]	√			√	√		√
Chang et al. [44]	√	√		√	√		
Chaim et al. [50]				√	√		√
Zhang et al. [43]	√		√		√		√
Li et al. [39]		√	√	√	√	√	√

**Table 2:** Indicators for industry–environmental system resilience evaluation.

Sub-Systems	Criteria Layer	Indicators	Direction
Industrial activity (A1)	Economy (B1)	Output value of the primary industry (C11) +	↓
		Output value of the secondary industry (C12) +	↓
		Output value of the tertiary industry (C13) ++	↑
	Production (B2)	Proportion of industry (C24) ++	↑
		industrial structure (C25) +	↑
		Output value of agriculture, Forest, Animal husbandry and fishery (C26) +	↓
Environment (A2)	Land resource (B3)	Construction land area (C37) *	↑
		cultivated field (C38) ++	↑
	Other resources (B4)	Energy consumption (C49) ++	↑
		Water consumption (C410) ++	↑
		Power consumption (C411) ++	↑
	Pollution (B5)	Wastewater discharge (C512) ++	↑
		Industry Waste gas (C513) ++	↑
		Industrial Smoke dust (C514) ++	↑
		Industrial Solid waste (C515) ++	↑
	Treatment (B6)	Industrial solid wastes utilization (C616) +	↓
		Wastewater treatment capacity (C617) ++	↑

++ denotes the highly cited indicators. + denotes the moderately cited indicators. * denotes the novel indicators.

**Table 3:** Different techniques for quantifying and modelling resilience.

Approaches	Measure	References	Description
Passive survival rate and proactive survival rate	*Resilience (Ψ) = Reliability (R) + Restoration (ρ)*	Youn, Hu and Wang [62]	Passive survival refers to the reliability of the system, and active survival represents the resilience of the system. Although this method is most suitable for earthquakes, it can still be used to quantify the resiliency of other systems.
Dynamic resilience (DR)	*DR* = $\overset{N}{\underset{i\;=\;1}{\sum }}SO_{HR}\left(t_{i}\right)-SO_{WR}(t_{i})$	Rose [63]	Dynamic resilience calculation based on hastened recovery (SO_HR_) and without hastened recovery (SO_WR_)
The Bayesian network	*P(Y1,Y2,Y3,…,Yn) = P(Y1/Y2,Y3,…,Yn)P(Y2, /Y3,…,Yn)…P(Yn-1/Pn)P(Yn)*	Fenton and Neil [64]	The BN is a powerful tool for risk evaluation, reliability prediction, and decision making under the stochastic conditions of a complex system. It makes statistical inference in a reasonable way by updating the prior beliefs of an elementary event
The catastrophe theory	Fold: *V = x*^3^ + *ax*; *Xa*_1_ = $\sqrt{a_{1}}$; Cusp: *V* = *x*^4^ + *ax*^2^ + *bx*; *Xa*_1_ = $\sqrt{a_{1}}$, *Xa*_2_ = $\sqrt[{3}]{a_{2}}$; Swallowtail: *V* = *x*^5^ + *ax*^3^ + *bx*^2^ + *cx*; *Xa*_1_ = $\sqrt{a_{1}}$, *Xa*_2_ = $\sqrt[{3}]{a_{2}}$, *Xa_3_* = $\sqrt[{4}]{a_{3}}$; Butterfly: *V* = *x*^6^ + *ax*^4^ + *bx*^3^ + *cx*^2^ + *dx*; *Xa*_1_ = $\sqrt{a_{1}}$, *Xa_2_* = $\sqrt[{3}]{a_{2}}$, *Xa_3_* = $\sqrt[{4}]{a_{3}}$, *Xa_4_* = $\sqrt[{5}]{a_{4}}$	Y. Li, Y.F. Li and M. Kappas [39]	The catastrophe theory contains four models with different equilibrium surfaces: Fold, Cusp, Swallowtail, and Butterfly. The dimension of control variables in a sub-system dictates the calculation model, which means the number of indicator in sub-system determines the model. Since catastrophe theory follows a hierarchical process, the resilience value is calculated by indicator to sub-system.

**Table 4:** Result of indicator data processing.

Year	Power Consumption	Water Consumption	Energy Consumption	Construction Land Area	Wastewater Discharge	Industry Waste Gas	Industrial Smoke Dust	Industrial Solid Waste
2000	0.471	0.066	0.233	0.716	0.694	0.402	0.95	0.274
2001	0.3973	0.0492	0.2133	0.6007	0.5325	0.3492	0.7736	0.2511
2002	0.3728	0.0536	0.2094	0.6587	0.5496	0.3363	0.7733	0.2299
2003	0.3873	0.0573	0.2105	0.681	0.5685	0.3301	0.7943	0.2231
2004	0.3748	0.06	0.1984	0.6098	0.5561	0.3031	0.8288	0.2145
2005	0.4644	0.0782	0.2305	0.7668	0.7103	0.3786	0.8045	0.2492
2006	0.4628	0.0699	0.2462	0.8122	0.5737	0.3838	0.3881	0.3265
2007	0.5887	0.0719	0.2409	0.8787	0.4723	0.5873	0.3146	0.2898
2008	0.6117	0.0815	0.2319	0.9023	0.6717	0.3943	0.2591	0.3086
2009	0.6189	0.0793	0.2169	0.8359	0.6916	0.3547	0.2862	0.2186
2010	0.6042	0.0811	0.1971	0.825	0.6663	0.3182	0.2428	0.1581
2011	0.5763	0.0813	0.1859	0.7925	0.6036	0.2463	0.1219	0.1127
2012	0.5476	0.082	0.1554	0.7326	0.6058	0.2489	0.1323	0.1176
2013	0.5343	0.0836	0.1492	0.7175	0.6323	0.147	0.1095	0.1059
2014	0.5406	0.0974	0.1806	0.7022	0.6454	0.1511	0.0938	0.0885
2015	0.5324	0.0904	0.187	0.7437	0.7108	0.1207	0.0855	0.0571
2016	0.545	0.1131	0.1822	0.8926	0.8141	0.1024	0.0756	0.0585
2017		0.1094	0.1748	0.8747	0.8405	0.0928	0.0743	0.0468

**Table 5:** Cluster range and resilience level of resilience values.

Grades	Resilience Interval
1. Non-resilience	<0.81
2. Low-resilience	0.81–0.82
3. Resilience	0.82–0.86
4. Mid-resilience	0.86–0.93
5. High-resilience	>0.93
